# Denoising of Optical Coherence Tomography Images in Ophthalmology Using Deep Learning: A Systematic Review

**DOI:** 10.3390/jimaging10040086

**Published:** 2024-04-01

**Authors:** Hanya Ahmed, Qianni Zhang, Robert Donnan, Akram Alomainy

**Affiliations:** 1 Department of Electronic Engineering and Computer Science, Queen Mary University of London, London E1 4NS, UK; 2 Department of Engineering and Materials Science, Queen Mary University of London, London E1 4NS, UK

**Keywords:** deep learning, ophthalmology, image processing, optical coherence tomography

## Abstract

Imaging from optical coherence tomography (OCT) is widely used for detecting retinal diseases, localization of intra-retinal boundaries, etc. It is, however, degraded by speckle noise. Deep learning models can aid with denoising, allowing clinicians to clearly diagnose retinal diseases. Deep learning models can be considered as an end-to-end framework. We selected denoising studies that used deep learning models with retinal OCT imagery. Each study was quality-assessed through image quality metrics (including the peak signal-to-noise ratio—*PSNR*, contrast-to-noise ratio—*CNR*, and structural similarity index metric—*SSIM*). Meta-analysis could not be performed due to heterogeneity in the methods of the studies and measurements of their performance. Multiple databases (including Medline via PubMed, Google Scholar, Scopus, Embase) and a repository (ArXiv) were screened for publications published after 2010, without any limitation on language. From the 95 potential studies identified, a total of 41 were evaluated thoroughly. Fifty-four of these studies were excluded after full text assessment depending on whether deep learning (DL) was utilized or the dataset and results were not effectively explained. Numerous types of OCT images are mentioned in this review consisting of public retinal image datasets utilized purposefully for denoising OCT images (*n* = 37) and the Optic Nerve Head (ONH) (*n* = 4). A wide range of image quality metrics was used; *PSNR* and *SNR* that ranged between 8 and 156 dB. The minority of studies (*n* = 8) showed a low risk of bias in all domains. Studies utilizing ONH images produced either a *PSNR* or *SNR* value varying from 8.1 to 25.7 dB, and that of public retinal datasets was 26.4 to 158.6 dB. Further analysis on denoising models was not possible due to discrepancies in reporting that did not allow useful pooling. An increasing number of studies have investigated denoising retinal OCT images using deep learning, with a range of architectures being implemented. The reported increase in image quality metrics seems promising, while study and reporting quality are currently low.

## 1. Introduction

Optical coherence tomography (OCT) stands at the forefront of modern medical imaging techniques, harnessing the power of low-coherence infrared light to delve deep into biological structures with unprecedented clarity and precision [1]. This revolutionary technology affords longer exposure times due to its inherent biological safety, presenting a stark departure from the ionizing radiation associated with conventional X-rays. Moreover, when juxtaposed with Magnetic Resonance Imaging (MRI) and Computerized Tomography (CT), OCT emerges as a cost-effective alternative, democratizing access to high-quality diagnostic imaging. However, amidst the brilliance of OCT lies a challenge inherent to all imaging modalities—noise. Inevitably introduced during the imaging process, noise mingles with the signal emanating from the object under scrutiny, influencing the resultant intensity observed by the detecting pixel [2]. Of particular concern is speckle noise, a byproduct of low coherence in irradiance, which casts a shadow over the signal-to-noise ratio, obscuring critical details within the imagery. Within the realm of ophthalmology, OCT serves as a versatile tool, facilitating the acquisition of cross-sectional and volumetric images that illuminate the intricate landscape of biological tissues and retinal structures. These images serve as invaluable aids in diagnosing a myriad of ocular diseases, ranging from diabetic retinopathy (DR) [3] to age-related macular degeneration (AMD) [4], guiding clinicians toward tailored treatment strategies.

Meanwhile, in the realm of computer science and Artificial Intelligence (AI), Machine Learning (ML) emerges as a beacon of innovation. By mining insights from past data, ML algorithms prognosticate future trends, obviating the need for explicit programming or human intervention [5]. At its core, ML epitomizes the essence of pattern recognition, endowing computers with the capacity to glean insights from vast datasets with unparalleled efficiency.

Within the ML landscape, deep learning (DL) commands center stage, propelled by leaps and bounds in computational prowess and the proliferation of “big data”. Convolutional Neural Networks (CNNs) epitomize this evolution, revolutionizing DL with their ability to extract features, classify images, and recognize patterns at breakneck speeds. By emulating the intricate workings of the human brain through the deployment of filters and intricate layers, CNNs herald a new era of computational efficiency.

The intersection of DL and OCT heralds a realm of boundless possibilities, marked by advancements in volumetric data handling, heightened sensitivity, and specificity in detecting structural alterations and the tantalizing prospect of denoising retinal images to unprecedented levels of clarity. This synthesis prompts a critical examination, as we delve into recent studies illuminating the applications of DL to OCT imagery, evaluating their impact on image quality assessment.

Moreover, as we stand on the precipice of a new era in medical imaging and computational innovation, it behooves us to explore the clinical ramifications of integrating these cutting-edge computational techniques into the fabric of healthcare delivery. Through a judicious examination of recent computational innovations and their potential clinical applications, we chart a course toward enhanced diagnostic accuracy, streamlined treatment pathways, and, ultimately, improved patient outcomes. Thus, this review not only serves as a testament to the symbiotic relationship between technology and healthcare but also as a compass guiding future research endeavors and clinical initiatives.

## 2. Overview of Optical Coherence Tomography

**Figure 1:**
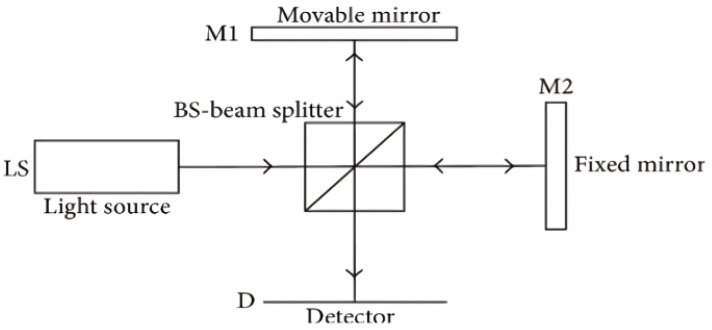
A system diagram of the principle of Michelson interferometry used in OCT [9]. The signal from a collimated light source (LS) is partitioned into two orthogonal beam paths by a beam splitter (BS); one collinear and the other normal to the ls pointing. The normal beam is reflected off a movable mirror (M1), the collinear off a fixed mirror (M2). The reflected beams are recombined at the bs and the co-propagating superposition is recorded as an interferogram at the detector (D). Image [9].

Optical coherence tomography (OCT) stands as a maturing imaging technology, offering resolution ranging from millimeters to sub-millimeters and boasting a penetration depth comparable to that achieved in human skin [6]. This innovative technique predominantly employs low-coherence infrared light to safely delve into biological tissues, affording longer exposure times in contrast to X-rays. Figure 1 illustrates the conceptual system configuration of Michelson interferometry, the foundation upon which OCT operates. Within this setup, the interferometric probe beam, formed by recombined reflected beams at the beam splitter, is directed toward the surface under examination, with a detector poised to capture the backscatter emanating from this surface [7].

Central to the functioning of OCT is the notion of coherence, a defining characteristic of light wherein all rays maintain a consistent and calculable phase over a defined period. However, the utilization of low coherence, while advantageous in probing biological tissues, introduces an unintended consequence—the introduction of noise altering pixel intensity and distorting the resulting image. This phenomenon manifests as artifacts, leading to a compromised signal-to-noise ratio within OCT images [7]; while various forms of noise may afflict the imaging process, speckle noise emerges as the predominant type encountered in OCT imagery [8].

At the heart of OCT lies the Michelson interferometer, serving as its primary setup. Here, an optical probe directs low-coherent light toward the sample, penetrating its surface and awaiting the rebound of reflected light. Subsequently, this reflected light is channeled to the interferometer via an optical fiber for meticulous analysis, as depicted in Figure 1 [9].

## 3. Material and Methods

This paper presents a systematic review focusing on studies concerning diagnostic accuracy. The reporting of this study adheres to the PRISMA-DTA guidelines [10].

### 3.1. Eligibility Criteria

This systematic review is designed to address specific queries utilizing the PICO framework, which encompasses Population, Intervention, Control, and Outcomes. Our investigation centers on several key questions. Firstly, we aim to explore the various implementations and accuracy outcomes associated with employing deep learning (DL) techniques for denoising retinal optical coherence tomography (OCT) imagery. Secondly, we seek to evaluate the effectiveness of these DL-based denoising models in mitigating noise and enhancing the quality of retinal OCT images. Thirdly, we will assess the performance of DL-based denoising models against other denoising methods or untreated OCT images used as benchmarks. Finally, our primary objective is to evaluate the effectiveness of DL-based denoising models by analyzing improvements in image quality through the application of widely used image metrics. Inclusion criteria for studies are as follows:

- Population (P): Studies focusing on the utilization of DL models with retinal imagery obtained from either clinical or research settings.
- Intervention and Control (I, C): Studies employing DL-based models for tasks such as image denoising, speckle reduction, or super-resolution, compared with a reference test.
- Outcomes (O): Studies reporting any estimate of image quality metrics (such as *PSNR*, *CNR*, *SSIM*) applied at either the image or pixel level.

Our exclusion criteria encompass studies that do not meet specific standards, including a lack of clear explanation regarding the utilized DL model, absence of an effective comparative analysis with other state-of-the-art denoising methods, and classification as reviews rather than original research contributions. By meticulously delineating these inclusion and exclusion criteria, our goal is to ensure the robustness and reliability of the systematic review findings. This approach facilitates comprehensive insights into the utility and efficacy of DL in enhancing the quality of retinal OCT imagery.

### 3.2. Search Methods for Identifying Studies

An electronic search was conducted within the following electronic databases: Google Scholar, Scopus, Medline (via PubMed), Embase, and ArXiv, covering entries up to 16 November 2023. Results were filtered to include publications from 2010 onward, as deep learning (DL) for computer vision and image analysis gained prominence following its development in 2012 by Krizhevsky et al. [11]. Language restrictions were not applied. Customized keywords were utilized for each database. Refer to Table 1 for the specific search queries employed. Moreover, additional studies were identified through screening conference proceedings and journal articles. Furthermore, manual cross-referencing of the bibliographies of included papers was performed to ensure comprehensive coverage.

### 3.3. Study Selection

To efficiently manage citations, BibTeX was employed as a tool. Initial screening involved the removal of duplicate entries based on titles and abstracts. Subsequently, a thorough evaluation of articles was conducted to identify eligible studies in accordance with the predetermined inclusion and exclusion criteria. This meticulous process ensured the selection of relevant and appropriate studies for the systematic review. Additionally, any discrepancies or uncertainties during the screening process were resolved through discussion among the research team, ensuring consistency and accuracy in study selection.

### 3.4. Data Collection and Extraction

Data collection was conducted independently from the included studies and meticulously revised to address any discrepancies or disagreements. Comprehensive information was extracted, encompassing various data items vital for analysis. These included bibliographic details such as authors’ names and publication years, details regarding the data modality and type of dataset utilized, hardware specifications, and dataset size (including train/validation/test sets, if provided). Moreover, inclusion and exclusion criteria at the image level, if available, were noted, along with the specified objective of the study (e.g., image denoising, speckle reduction, super resolution). Information regarding pre-processing techniques, data augmentation strategies, and the deep learning (DL) approach employed, including the neural network (NN) architecture utilized, was also recorded. Additionally, details regarding the loss function employed and the image quality metrics used for evaluation were documented. The resulting findings from each study were thoroughly examined. In cases where an article compared multiple NN architectures, the most accurate one was reported to ensure clarity and consistency in the analysis. This comprehensive approach to data extraction facilitated a robust and thorough examination of the included studies, contributing to the reliability and validity of the systematic review findings.

### 3.5. Risk of Bias and Applicability

This review focuses specifically on the denoising aspect, and to assess the risk of bias in the included studies, we adapted and employed the QUADAS-2 tool. This modified tool encompasses four main domains addressing the risk of bias: the data selection, index test, reference standard, and flow-and-timing. Additionally, it evaluates three domains regarding the applicability of the study to patient selection, the index test, and the reference standard.

Within the “data selection” domain, we scrutinized papers with vague data-split strategies and limited information on the dataset, which could potentially lead to data leakage, indicating a high risk of bias. Moving on to the “index test” domain, we assessed indicators such as the lack of description of the model and the absence of details regarding test recreation and reproducibility. The “flow-and-timing” domain was evaluated based on indicators such as the implementation of multiple reference standards (i.e., state-of-the-art denoisers) within the same article and the appropriateness of intervals between the index test and reference standard. Finally, within the “reference standard” domain, we considered indicators like inadequate information on reference standard definition and the utilization of only one reference test.

In cases where concerns arose regarding the relevance of the studies, certain factors were meticulously reviewed. These included the dataset used, the procedure employed for creating clean data, the specific deep learning (DL) model utilized, and its performance concerning image quality metrics. Table 2 outlines the key questions utilized in our assessment process, providing a structured framework for evaluating the risk of bias across the included studies. Through this comprehensive approach, we aimed to ensure the rigor and reliability of our review findings.

### 3.6. Data Synthesis and Analysis

Due to the diverse array of study designs and image quality measures utilized, our quantitative synthesis was primarily confined to examining the outcomes related to image denoising and speckle reduction. Given the substantial variability in the image quality metrics employed for quantifying denoising and super resolution, the scope for direct comparison was somewhat constrained, while a minority of studies reported metrics such as contrast-to-noise ratio (*CNR*), equivalent number of looks (*ENL*), and the structural similarity index measure (*SSIM*), the predominant metrics presented were peak signal-to-noise ratio (*PSNR*) or signal-to-noise ratio (*SNR*). Consequently, conducting a comparative quantitative analysis across studies was limited to reporting any or all evaluation metrics mentioned, calculated by:

(1) $$PSNR=10\log(\frac{L^{2}}{MSE}),$$

(2) $$SNR=10\log(\frac{P_{signal}}{P_{noise}}),$$

*L* denotes the maximum possible pixel value, and MSE is the mean squared error of the image. $P_{signal}$ and $P_{noise}$ are the mean and standard deviation of pixel values, respectively. Next, the structural similarity index (*SSIM*) is a well-known image quality metric that focuses on perceived similarity. The *SSIM* focuses on texture, quality degradation and visible structures. The *SSIM* is defined as

(3) $$SSIM=\frac{\left(2\sigma_{nc}+c_{2}\right)\left(2\mu_{n}\mu_{c}+c_{1}\right)}{\left(\mu_{n}^{2}+\mu_{c}^{2}+c_{1}\right)\left(\sigma_{n}^{2}+\sigma_{c}^{2}+c_{2}\right)}$$

$\mu_{n}$, $\mu_{c}$, and $\sigma_{n}$, $\sigma_{c}$ are the mean value and standard variation in noisy (n)–clean (c) image pairs, respectively. Lastly, contrast-to-noise ratio (*CNR*) utilizes ROIs of background and signal areas for speckle repression with respect to both areas. Equivalent number of looks (*ENL*) is a metric assessing the smoothing of the predicted image. It does not require a reference image since it utilizes selected ROIs of background and signal. *ENL* is defined as

(4) $$ENL=\frac{\mu_{b}^{2}}{\sigma_{s}^{2}}$$

$\sigma_{s}$ is the standard deviation of the signal representation, and $\mu_{b}$ is the mean value for background representation. *CNR* is calculated through

(5) $$CNR=10\log(\frac{\mu_{s}-\mu_{b}}{\sqrt{\sigma_{b}^{2}+\sigma_{s}^{2}}})$$

$\mu_{s}$ and $\sigma_{s}$ are the mean value and standard deviation of the signal representation, respectively. For background representation, $\mu_{b}$ and $\sigma_{b}$ are the mean value and standard deviation.

## 4. Results

### 4.1. Study Selection and Study Characteristics

**Figure 2:**
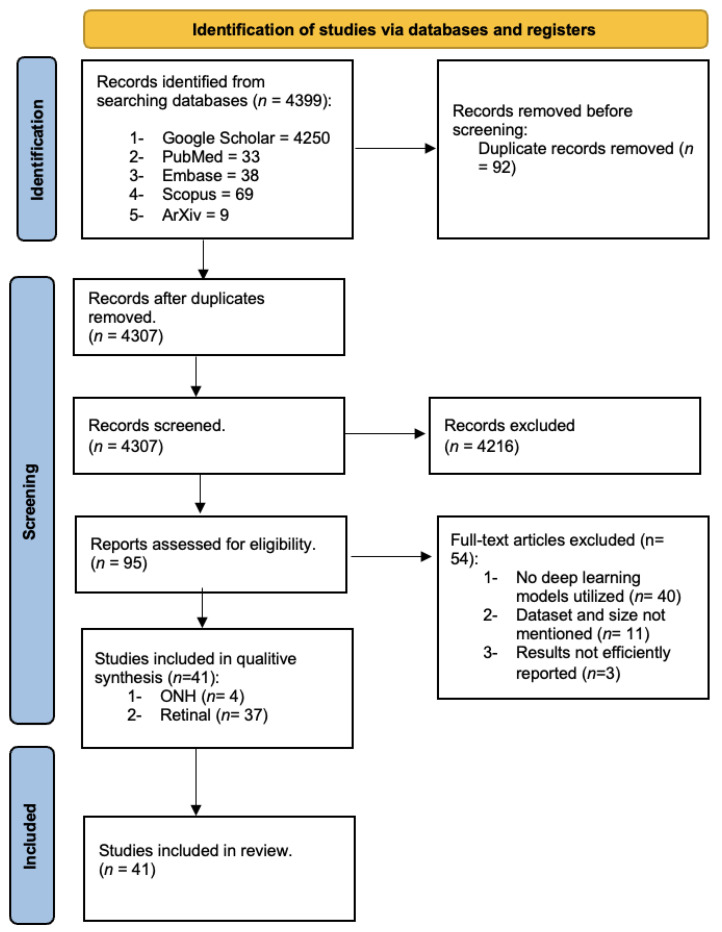
Flowchart of the search following the PRISMA guidelines.

Out of the initial pool of 4399 studies identified, a rigorous evaluation was conducted for 41 studies based on the criteria outlined in Table 2, utilizing their full texts. Subsequently, 54 studies were excluded following a thorough assessment of their full texts. The reasons for exclusion were carefully documented and categorized, with detailed explanations provided in Table S1. Ultimately, after meticulous manual screening, a total of 41 studies were deemed eligible for inclusion in our review. Notably, the number of studies included per year exhibited an upward trend over the observation period, as depicted in Figure 2. This trend underscores the increasing interest and attention devoted to the subject matter over time, highlighting the evolving landscape of research in this field.

The studies examined in this review were categorized into two main groups based on the type of optical coherence tomography (OCT) images utilized within the field of ophthalmology: Optic Nerve Head (ONH) images and retinal images. A summary of these studies is presented in Table 3 and Table 4, respectively, highlighting the utilization of various deep learning (DL) models for tasks such as image denoising, speckle reduction, and super resolution. Specifically, retinal image datasets utilized in this review were predominantly public datasets purposely employed for denoising OCT images (*n* = 37). Notable datasets included DUKE [12], Topcon [13], OPTIMA [14], Cirrcus [15], and Heindberg [16]. Conversely, ONH images (*n* = 4) primarily comprised private datasets created by researchers.

The majority of studies (*n* = 39) incorporated multiple reference tests to evaluate their proposed methods against previous state-of-the-art denoisers. These reference tests encompassed both traditional programming and DL denoising models. Specifically, 26 studies implemented DL models and traditional programming as reference tests, while 9 studies utilized traditional programming-based state-of-the-art denoisers such as BM3D and NLM. However, five studies did not specify any established reference test.

**Figure 3:**
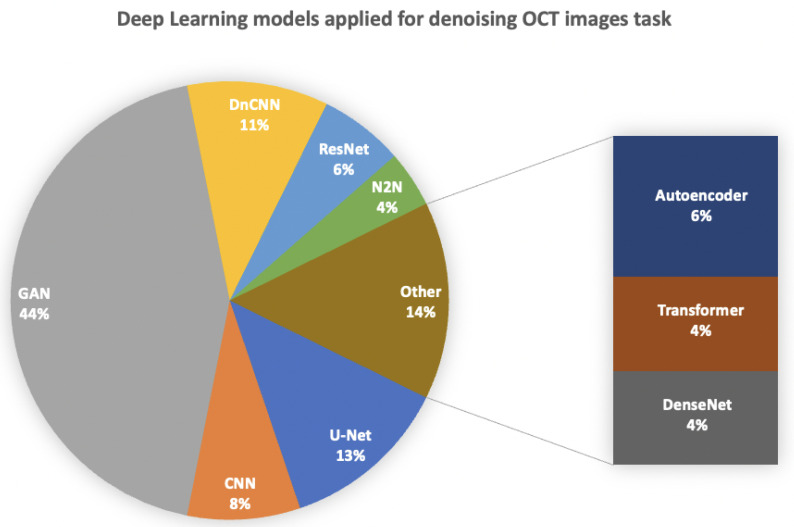
Deep learning models approaches involved for denoising OCT images (*n* = studies).

Regarding the choice of dataset for denoising, the DUKE dataset (*n* = 22) was the most frequently utilized, followed by Topcon (*n* = 6), with ONH images being the least employed (*n* = 4). Various DL models were deployed and integrated into hybrid frameworks, as depicted in Figure 3, which illustrates the distribution of studies implementing each DL model. Notably, 44% of the studies implemented a hybrid generative adversarial network (GAN), with the super-resolution GAN (SR-GAN) being the most utilized (*n* = 3) alongside the conditional GAN (cGAN) (*n* = 3). Additionally, 13% of the studies employed a traditional U-Net model (*n* = 6) for denoising, with only one hybrid framework reported.

Evaluation of denoising, speckle reduction, and studies on super resolution primarily relied on metrics such as *PSNR* and *SNR* as the major image quality indicators. Furthermore, other widely used image quality metrics included the *SSIM* (*n* = 27), *CNR* (*n* = 24), and *ENL* (*n* = 16). These metrics are comprehensively displayed in Table 3 and Table 4, providing insight into the methodologies and outcomes of the reviewed studies.

### 4.2. Risk of Bias and Applicability

Every study included in the review underwent a thorough assessment of risk of bias, with the results meticulously documented in Table S2. Out of the studies evaluated, 10 (27%) were identified as having a low risk of bias across all four domains. Notably, the domain presenting the most challenges was the “index test”, with only 22 studies (59.5%) categorized as having a low risk of bias in this area. This finding underscores the importance of critically evaluating the methodology and execution of the index test within each study to ensure the reliability and validity of the findings. Through this rigorous risk-of-bias assessment, we aimed to provide a comprehensive evaluation of the methodological robustness of the included studies, thereby enhancing the credibility and trustworthiness of our review outcomes.

### 4.3. Findings of the Studies

When focusing on peak signal-to-noise ratio (*PSNR*) and signal-to-noise ratio (*SNR*) as metrics for denoising images, a significant variation in deep learning (DL) techniques across different types of optical coherence tomography (OCT) images was observed. Specifically, for studies utilizing Optic Nerve Head (ONH) images, *PSNR* or *SNR* values ranged from 8.1 to 25.7 dB, while, for retinal datasets, these metrics ranged from 26.4 to 158.6 dB.

The majority of studies predominantly applied public retinal datasets such as Duke [12], Topcon [13], and Cirrus [15], primarily due to their extensive availability and widespread use. However, it is noteworthy that these datasets typically do not provide “clean” versus “noisy” image pairs required for denoising tasks. Consequently, researchers resorted to generating clean images through alternative means. For instance, clean images were generated by averaging multiple B-scans or employing traditional programming techniques. In some cases, unsupervised DL techniques were also utilized to generate clean images. These approaches aimed to provide a reliable basis for assessing the effectiveness of DL-based denoising methods, despite the absence of explicitly labeled clean and noisy image pairs within the datasets.

## 5. Discussion

Numerous DL tasks consisting of detection, segmentation and classification are challenging in ophthalmology since OCT is the main imaging technique in that specific field. OCT introduces speckle noise that adds artifacts, thereby impairing image quality and confounding correct clinical interpretation [59]. Thus, DL has provided solutions for denoising and speckle reduction in OCT images to overcome this problem. In this systematic review, many studies were compiled and analyzed to assess the application of DL for image denoising of OCT images in ophthalmology. Over the past ten years, multiple researchers have shown a developing and promising body of evidence supporting DL for this task. Even so, there was a limited supply of quality studies for comparison across traditional programming and DL. A number of findings require more detailed discussion. The challenge of addressing numerous deep learning (DL) tasks in ophthalmology, including detection, segmentation, and classification, is compounded by the prevalent use of optical coherence tomography (OCT) as the primary imaging technique in this field. OCT introduces speckle noise, which can adversely affect image quality, thereby complicating accurate clinical interpretation. DL techniques have emerged as promising solutions for denoising and reducing speckle artifacts in OCT images, with this systematic review analyzing a multitude of studies to assess the application of DL for image denoising in ophthalmology. Over the past decade, researchers have generated a growing body of evidence supporting the efficacy of DL for this purpose. As shown in Figure 3, the main DL models implemented were the GAN, DnCNN, Autoencoder, U-Net, Noise2Noise, and transformer, etc.

Generative adversarial networks (GANs) exhibit promise in synthesizing synthetic OCT images with reduced noise levels. By training a generator network to generate realistic images and a discriminator network to differentiate between real and generated images, GANs have demonstrated their capability to denoise OCT scans effectively while preserving clinically relevant features. However, GANs can be challenging to train and prone to mode collapse, where the generator produces limited variations in images, potentially limiting their diversity and generalizability in clinical settings. In contrast, Denoising Convolutional Neural Networks (DnCNNs) utilize deep convolutional layers to understand the underlying structure of noisy OCT images and produce clean counterparts. Through iterative training on paired noisy–clean image datasets, DnCNNs efficiently enhance image clarity. However, DnCNNs may struggle with complex noise patterns and require large amounts of labeled data for training, which may be resource-intensive and time-consuming to acquire. The U-Net architecture, characterized by its symmetric encoder–decoder structure with skip connections, has showcased remarkable performance in semantic segmentation tasks, including the denoising of OCT images. By integrating contextual information from various spatial scales, U-Net effectively preserves anatomical structures. Nonetheless, U-Net architectures may suffer from memory inefficiency and computational overhead, particularly when handling high-resolution OCT images. Autoencoders, consisting of an encoder and decoder network, learn to reconstruct input data from a compressed representation. Trained on noisy OCT images, autoencoders can encode essential features suitable for clinical interpretation. However, autoencoders may struggle with capturing complex image structures and may require careful tuning of hyperparameters to achieve optimal performance. Transformers, initially designed for natural language processing tasks, have recently been applied in image processing, including denoising. Leveraging self-attention mechanisms to capture long-range dependencies, transformers effectively preserve spatial information in OCT images. However, transformers may suffer from scalability issues when applied to large-scale image datasets due to their computational complexity and memory requirements. The Noise2Noise approach involves training deep learning models directly on pairs of noisy images, eliminating the need for clean reference images during training. By exploiting inherent redundancies in noisy data, Noise2Noise denoises OCT images without access to ground truth clean images, making it suitable for real-world clinical applications where clean reference images may not be readily available. Nevertheless, Noise2Noise may struggle with highly variable noise patterns and may require careful regularization techniques to prevent overfitting to noise artifacts.

These innovative DL techniques have the potential to revolutionize clinical workflows in ophthalmology by automating the denoising process and improving the efficiency and accuracy of OCT image interpretation. By providing clinicians with high-quality, denoised images, these techniques can facilitate more confident diagnoses and treatment decisions, ultimately leading to improved patient outcomes. However, the scarcity of high-quality studies for comparison across traditional programming and DL remains limited, necessitating a closer examination of key findings.

Firstly, a significant proportion of studies (62%) conducted their DL model training and testing on public datasets, validating their reported image quality metrics and comparing them with other state-of-the-art DL models (reference tests). These studies demonstrated relatively high *PSNR* or *SNR* values for denoised images, indicating substantial improvement in image quality attributable to DL models. Given the considerable noise inherent in OCT images, DL techniques hold significant promise for enhancing diagnosis, segmentation, and detection tasks.

Secondly, only studies utilizing the Duke [12] public dataset had access to clean versus noisy image pairs for training purposes. Other studies, utilizing datasets like Topcon [12] and Cirrus [15], lacked clean images and thus resorted to creating their own algorithms or applying traditional programming techniques to obtain “ground truths” for their B-scans. This variance in ground truth generation methods complicates cross-study comparisons and underscores the importance of clearly outlining and validating strategies for creating clean images to ensure the production of robust models.

Thirdly, there was considerable variability in both the reporting and conduct of the studies, posing challenges for comprehensive quantitative and qualitative analyses across the board. Given the wide array of available image quality metrics beyond *PSNR* or *SNR* (such as *CNR*, *ENL*, and *SSIM*), it was observed that studies typically reported only two or three metrics. This lack of uniformity in reporting was further exacerbated by the implementation of multiple and varied reference tests. For instance, several studies utilized a diverse range of reference tests, resulting in a vague impact assessment on either *PSNR* or *SNR*. Consequently, there is a pressing need for a standardized structure for image denoising studies, encompassing specific reference tests (including the appropriate balance of traditional programming and deep learning models) and a predetermined set of specified image quality metrics (such as *PSNR*, *SSIM*, *CNR*, and *ENL*). Consequently, the feasibility of conducting a meta-analysis was limited, as only a small subset of studies provided similar image quality metrics beyond *PSNR* or *SNR*. This situation underscores the significant consequences stemming from the lack of minimum standards and tools available in the field of image denoising, where diagnostic studies are currently limited to utilizing only two tools (STRAD-AI and QUADS-2). Since the studies included in this review solely reported either *PSNR* or *SNR* of the denoised images in comparison to multiple reference tests, it remains uncertain whether DL provides significant assistance in image denoising and speckle reduction. Ideally, the impact of DL for denoising OCT images in ophthalmology should be demonstrated in practice-based settings and validated by its ability to improve further objectives such as detection [60], classification [61], and segmentation [62], which the majority of included studies did not consider.

Fourthly, a significant proportion (74%) of papers were excluded from the review due to the absence of DL methodologies, instead focusing on improving traditional programming methods such as wavelets and shearlets [63], NLM [64], and BM3D [65]. This suggests that DL has not been extensively investigated for OCT image denoising. The remaining papers were excluded due to inadequate descriptions of datasets and their utilization, as well as a lack of reference testing to demonstrate their impact on *PSNR* and *SNR*.

Fifthly, and perhaps most importantly, none of the studies incorporated input from clinicians regarding the produced results. This is particularly concerning considering that the primary purpose of denoising OCT images is to assist clinicians in diagnosing various retinal diseases. Ideally, clinicians should provide feedback on the reported denoised images, assessing whether useful data has been removed or added that could significantly impact diagnosis accuracy.

Lastly, it is essential to emphasize that OCT is the primary instrument in ophthalmology for capturing multiple types of images (including ONH and retinal images) crucial for detecting retinal diseases. This systematic review has revealed both strengths and limitations, with a systematic and extensive assessment of studies conducted to compare DL for image denoising, speckle reduction, and super resolution for OCT images in ophthalmology; while there was a restricted timeline justified by the focus on DL, a substantial and diverse body of evidence has been presented, validating the necessity for such limitations. Additionally, the absence of a meta-analysis was attributed to the mixed nature of reporting and the lack of quality in comparative result analysis.

Therefore, there has been a growing number of studies investigating the denoising of OCT images in ophthalmology using deep learning, with various computational architectures being explored. Among the reported metrics of image quality, the peak signal-to-noise ratio (*PSNR*) has emerged as a reliable metric for intercomparison, with values spanning from 8.1 to 25.7 dB for ONH images and 26.4 to 158.6 dB for retinal datasets. Moving forward, it is imperative for future studies to clearly outline reference tests and datasets, relying on a common, extensive, and clinically meaningful outcome basis to drive progress in the field.

## 6. Conclusions

In summary, the landscape of research in denoising Optical Coherence Tomography (OCT) images within ophthalmology has seen a notable surge, with a diverse array of studies delving into the application of deep learning techniques. This exploration has encompassed various computational architectures, reflecting a dynamic and evolving field seeking optimal solutions for image enhancement. Among the array of metrics used to evaluate image quality, the peak signal-to-noise ratio (*PSNR*) has emerged as a consistent benchmark for intercomparison, offering insights into the effectiveness of denoising methodologies. Notably, *PSNR* values have exhibited substantial ranges, underscoring the complexity and variability inherent in OCT image denoising efforts. Looking ahead, it is essential for future investigations to prioritize transparency and standardization in methodologies, particularly in outlining reference tests and datasets. By establishing a common foundation grounded in clinically meaningful outcomes, researchers can foster more robust advancements and ensure the translation of findings into tangible benefits for clinical practice and patient care.

## Figures and Tables

**Table 1:** The results of the electronic search in multiple databases.

Database	Keywords	Results	Date
Google Scholar	Image denoising AND deep learning AND “optical coherence tomography”	4220	16 November 2023
Medline	Image denoising AND deep learning AND “optical coherence tomography”	32	16 November 2023
Scopus	TITLE-ABS-KEY (image AND denoising AND deep AND learning AND “optical coherence tomography”)	68	16 November 2023
Embase	(“image denoising”/exp OR “image denoising” OR ((“image”/exp OR image) AND (“denoising”/exp OR denoising))) AND (“deep learning”/exp OR “deep learning” OR (deep AND (“learning”/exp OR learning))) AND (“optical coherence tomography”/exp OR “optical coherence tomography”)	37	16 November 2023
ArXiv	Image denoising AND deep learning AND “optical coherence tomography”	9	16 November 2023

**Table 2:** Modified leading questions of QUADAS-2 for critical appraisal.

Domain	Leading Questions
Data Selection	1—Are any data imbalances addressed in the article? 2—Was the dataset split explained correctly for training, validation, and testing? 3—Did the study collect sufficient noisy–clean image pairs?
Index test	1—Was the methodology sufficiently explained for reproducibility? 2—Were the results of deep learning models explained without knowledge of state-of-the-art denoisers? 3—Did the study apply any image quality metrics, sensitivity or robustness analysis on their model?
Flow and Timing	1—Was the full dataset utilized in the analysis? 2—Did each image have a reference clean image? 3—Were the reference clean images produced similarly? 4—Did the model show a sufficient interval between the reference and index test?
Reference Standard	1—Were state-of-the-art results of denoisers mentioned and utilized for interpretation? 2—Did the study describe the noisy–clean image procedure and minimize bias? 3—Were limitations, biases, and generalization issues reported sufficiently?

**Table 3:** Summary of findings in the selected studies utilizing Optic Nerve Head (ONH) datasets for image denoising, speckle reduction, and super-resolution.

Paper	Data Size	Pre-Processing and Augmentation	Model	Compared to	Loss Function	Image Quality Metrics
Devalla, 2019 [17]	24,832 B-Scans (23,280/1552)	Augmentation: Rotation, flip, elastic deformation Pre-Processing: NA	U-Net with residual blocks	NA	MAE	SNR = 8.14 dB CNR = 7.63 dB MSSIM = 0.65
Cheong, 2021 [18]	2628 B-Scans (2328/300)	Augmentation: rotation, translation, flip, and scaling Pre-Processing: NA	Hybrid model (framework containing U-Net followed by a choice of ResNet, ResXNet, or EfficientNet)	NA	Shadow + content + style	PSNR = 11.1% AGM = 57.2% CNR = 154% SSIM = 187%
Tian, 2020 [19]	350 HQ scans (315/35)	Augmentation: flip, rotation, cropping	RMA-GAN	SRCNN, EDSR, ESRGAN	Content + perceptual + adversarial + MS-SSIM + TV	PSNR = 25.7 dB SSIM = 0.77
Hu, 2020 [20]	2500 B-Scans	Augmentation: NA	MSUN with self-fusion	NA	L1 + MSE	PSNR = 10.1 dB SSIM = 0.57
Akter, 2020 [21]	157 B-Scans (100/57)	Augmentation: NA Pre-processing: contrast adjustment, sharpening filter, manually removed noise using Fiji: ImageJ	U-Net	WIN5-RB, Autoencoder, DnCNN, Dense-UNet	MSE	PSNR = 29.8 dB SSIM = 0.90 MSE = 0.005 MAE = 0.03
Halupka, 2018 [22]	55,080 B-Scans (40,711/5587/ 8780)	Augmentation: flip, rotation, cropping	GAN	WGAN, BM3D, DD-CDWT	MSE + VGG + adversarial	PSNR = 32.3 dB SSIM = 0.78 MSE = 40.3 MS-SSIM = 0.92

Keywords: MSE—Mean squared error; MAE—Mean absolute error; SNR—Signal-to-noise ratio; PSNR—Peak signal-to-noise ratio; CNR—Contrast-to-noise ratio; SSIM—Structural similarity index measure; MS-SSIM—Multi-scale structural similarity index measure; RMA-GAN—Realistic mixed attention GAN; SRCNN—Super-resolution CNN; EDSR—Enhanced deep super-resolution network; ESRGAN—Enhanced super-resolution GAN; MSUN—Multi-scale U-Net; WGAN—Wasserstein GAN.

**Table 4:** Summary of findings in the selected studies utilizing retinal datasets for image denoising, speckle reduction, and super-resolution.

Paper	Data Size	Pre-Processing and Augmentation	Model	Compared to	Loss Function	Image Quality Metrics
Wei, 2018 [23]	Duke [12] 26 B-Scans (22/4)	Pre-processing: KNN for clustering Augmentation: scaling	DnCNN	BM3D, NLM, BM3D-SAPCA, LPG-PCA, Low Rank, FFDnet	NA	PSNR = 28.2 dB CNR = 3.9 dB MSR = 6.2
Chen, 2020 [24]	36 B-Scans (25/11)	Pre-processing: aligning, averaging, thresholding, adding speckle noise	DN-GAN	MSBTD, SBSDI, BM3D, K-SVD, Tikkhonov, SRResNet, GAN-SRResNet, DCSRN, GAN-U-Net	L1 + perceptual	PSNR = 27.9 dB SSIM = 0.9 FBE = 3.6
Gour, 2020 [25]	Duke [12] and Topcon [13], 23 B-Scans	NA DnCNN	Adaptive Median Filtering, wavelet thresholding, Tikhonov, BM3D, K-SVD, MSBTD, Anisotropic diffusion, STAT, Bayesian, Isotropic diffusion, SE-CNN	MSE	PSNR = 27.5 dB SSIM = 0.68	
Hassan, 2021 [26]	10,000 B-Scans (8000/2000)	Pre-processing: added speckle noise	D-GAN	Wavelet, Bilateral, NLM, BM3D	Euclidean + perceptual + adversarial	PSNR = 35.4 dB MSE = 0.19
Ma, 2018 [15]	Duke [12] and Topcon [13] 521 B-Scans (512/9)	Pre-processing: registration, alignment and enhancing contrast Augmentation: flip, scaling, rotation, non-rigid transformation	cGAN	NLM, BM3D, STROLLR, K-SVD, MAP, DnCNN, ResNet	MSE + L1 + edge	SNR = 60.1 dB CNR = 10.0 dB ENL = 126.9 dB EPI = 1.0
Guo, 2020 [27]	Duke [12], A2A SD-OCT, 90 B-Scans (10/80)	NA	Nonlocal GAN	NLM, BM3D, K-SVD, BM4D, GCBD, GAN-MSE, DnCNN, GAN-WDP, DeGAN	Binary cross-entropy	SNR = 40.1 dB ENL = 981.3 dB CNR = 7.4 dB
Qiu, 2020 [28]	47 B-scans (37/10)	Pre-processing: averaged and registered the B-scans to create denoised image pairs	DnCNN	NLM, BM3D	Perceptually sensitive (SSIM loss)	PSNR = 26.4 dB SSIM = 0.71 MSE = 89.6 MS-SSIM = 0.91
Huang, 2021 [13]	OCT2017 [29], 84,500 B-scans (83,416/32/968)	Augmentation: crop	AC-SRResNet	BM3D, U-Net, SRResNet	L1	SNR = 41.8 dB CNR = 44.6 dB EPI = 0.72
Halupka, 2018 [22]	69 OCT volumes (51/7/11)	Pre-processing: averaged and registered the B-scans to create denoised image pairs	GAN	BM3D, DD-CDWT, CNN-WGAN	Adversarial + MSE + perceptual	PSNR = 32.3 dB SSIM = 0.78 MS-SSIM = 0.92 MSE = 40.3
Qiu, 2021 [30]	Duke [12], 52 groups of 50 B-scans each (37/15)	NA	P2PGAN-N2N	Median, NLM, BM3D	Adversarial + L1	SNR = 35.5 dB SSIM = 0.81 CNR = 4.0 dB ENL = 260.3 dB R = 0.94
Abassi, 2019 [31]	Duke [12], 28 B-Scans (10, 18)	Augmentation: flip, rotate, crop	MIFCN	KSVD, BM3D, SAIST, PG-GMM, BM4D, SSR	MSE	PSNR = 27.4 dB CNR = 3.8 dB ENL = 2750.8 dB
Shi, 2019 [32]	Topcon [13] and Cirrus [15], 11 groups of 256 B-Scans (2/9)	NA	DeSpec-Net	NLM, BM3D, STROLLR, K-SVD, MAP, Intra-volume compounding, DnCNN	L1	SNR = 40.2 dB CNR = 9.7 dB ENL = 166.2 dB EPI = 0.91
Huang, 2020 [33]	Duke [12], 26 B-scans (10/16)	Pre-processing: registering and averaging images, removing any over smoothed images	DRGAN	Median, Bilateral, NLM, Wavelet, BM3D, SNR-GAN, NWSR, edge-sensitive cGAN, HDCycleGAN, Nonlocal GAN, SiameseGAN	Adversarial + reconstruction + cycle-consistency + novel noise	PSNR = 24.4 dB SSIM = 0.58 CNR = 3.2 dB EPI = 0.98 MSR = 4.8 ENL = 317.4 dB
Yu, 2018 [34]	Duke [12], 15 B-scans (8/3/4)	Pre-processing: crop, removing unaligned images	DN-GAN	BM3D, BM3DPCA, LPGPCA, FFDNET	MSE + adversarial	PSNR = 31.0 dB CNR = 3.3 dB MSR = 3.7
Tajmirr-iahi, 2021 [35]	Topcon [13], 240 B-Scans (200/40)	Augmentation: rotation, shift, flip, and crop	Autoencoder	GT-SC-GMM, BM3D, MSBTD, Tikhonov	MSE	SNR = 108.8 dB CNR = 82.2 dB ENL = 58.4 dB TP = 0.79 EP = 0.98 CT = 4.68
Sengupta, 2021 [36]	Duke [12], 1600 B-Scans (1400/200)	Pre-processing: crop	EdgeWaveNet	NLM, DeBlur-GAN, RDNSR-GAN, RED-GAN	L1 + adversarial + Sobel edge	PSNR = 22.8 dB SSIM = 0.61
Mehdi-zadeh, 2021 [37]	71 B-scans (51/20)	Augmentation: created patches	DnCNN	NA	L2 + L1 + perceptual + VGG	PSNR = 33.6 dB PSI = 0.23 JNB = 13.9 S3 = 0.26
Cai, 2018 [38]	Topcon [13], 256 B-scans (246/10)	Pre-processing: averaged and registered the B-scans to create denoised image pairs	ResNet	Median, NLM, BM3D	MSE	PSNR = 34.8 dB SSIM = 0.52
Zhou, 2022 [39]	5000 B-scans (4500/480/20)	Pre-processing: crop	Transformer-IP2	BM3D, PNLM, NCDF, OBNLM, DnCNN, CNN-NLM, Neighbor2Neighbor	Neighbor- 2Neighnor + PNLM	SNR = 154.6 dB CNR = 7.9 dB ENL = 13,160.3 dB
Anoop, 2021 [40]	Duke [12] and Optima [14], 2720 B-scans (2176/544)	Pre-processing: noise distribution is found for each image, patches and denoised image pairs were created	DenseNet121	CAD, OBNLM, TVG, Wavelet, K-SVD, DnCNN	Cross-entropy	PSNR = 31.0 dB SSIM = 0.91
Fu, 2021 [41]	Duke [12], 21 B-scans (16/5)	Pre-processing: registering and averaging images, removed any over smoothed images	ADGAN	Wavelet, NLM, BM3D, NWSR, HDCycleGAN	Adversarial + cycle-consistency	PSNR = 27.6 dB SSIM = 0.62 CNR = 3.1 dB ENL = 530.8 dB
Wang, 2021 [42]	Topcon [13] and Cirrus [15], 1920 B-scans (512/1408)	Pre-processing: creating denoised image pairs from [23]	Capsule cGAN	BM3D, K-SVD, NLM, MAP, STROLLR, DnCNN, ResNet, Cycle-GAN, cGAN	L1 + Adversarial + SSIM	SNR = 59.0 dB CNR = 11.4 dB ENL = 417.2 dB EPI = 1.0
Zhou, 2022 [43]	Topcon [13] and Cirrus [15], 1920 B-scans (512/1408)	Pre-processing: registering and averaging images	Cycle-GAN with mini-cGAN	NLM, BM3D, STROLLR, K-SVD, MAP, DnCNN, DPDNN, NAGAN with mini-cGAN	L1 + MSE	SNR = 20.9 dB CNR = 12.5 dB SSI = 0.09 EPI = 0.99
Wu, 2021 [44]	3737 B-scans, (3537/200)	Pre-processing: crop and contrast enhancement	cGAN	Cycle-GAN, DnCNN, BM3D, DCWT, NLM, MPE, cGAN, EGAN, SR	Adversarial + cycle-consistency + structural consistency + regularization	SNR = 35.0 dB CNR = 7.2 dB EPI = 0.92 CRSB = 0.14
Das, 2020 [45]	Duke [12] 45 B-scans and 384 OCT volumes, (2000/17)	Pre-processing: crop	SRGAN	SBSDI, SSR, NWSR, SRGAN	Adversarial + cycle-consistency + identity mapping	PSNR = 39.2 dB CNR = 4.7 dB
Huang, 2019 [46]	Duke [12], 26 B-scans (10/16)	Pre-processing: crop	SDSR-OCT	BM3D + Bicubic, NWSR, SRCNN	Pixel + perceptual + GAN	PSNR = 28.1 dB CNR = 4.6 dB ENL = 537.5 dB EPI = 0.95
Ge, 2022 [47]	Duke [12], 10 B-scans	Pre-processing: clear images are obtained by registering and averaging and crop	Self2Self-OCT	BM3D, NWSR, DnCNN, DIP, TSI	Background noise attenuation + self-prediction	PSNR = 24.8 dB SSIM = 0.99
Ma, 2022 [48]	Duke [12], 26 B-scans (10/16)	NA	DSGAN	MIFCN, Edge-sensitive cGAN, SDSR-OCT	Adversarial + SSIM + MSE	PSNR = 28.1 dB SSIM = 0.95 CNR = 3.7 dB
Xie, 2022 [49]	Duke [12], 26 B-scans (22/4)	NA	GAN	K-SVD, BM3D, wGAN, cGAN, SDSR, HDcycleGAN, DRGAN	Adversarial + cycle-consistency + perceptual	PSNR = 27.6 dB EPI = 1.0 CNR = 3.1 dB ENL = 73.8 dB MSR = 5.1 SSIM = 0.65
Xie, 2023 [50]	Duke [12], 26 B-scans (10/16)	NA	MGAN	NLM, BM3D, DnCNN, MIFCN, SDSR-OCT	Adversarial + pixel-level error + BCE+ SSIM	PSNR = 28.1 dB SSIM = 0.95 EPI = 0.99 CNR = 3.6 dB
Ahmed, 2022 [51]	Duke [12], 18 B-scans (10/8)	Pre-processing: clean images are obtained by BM3D, BM3DDEB, Weiner and HWT	DenseNet with AG	BM3D, NLM	MSE + pixel difference	PSNR = 23.5 dB CNR = 7.7 dB ENL = 585.5 dB
Ahmed, 2022 [52]	Duke [12] and dentistry, 28 B-scans (18/12)	NA	Autoencoder with MFSK and AG	BM3D, NLM, DnCNN, GAN	MSE + pixel difference	PSNR = 26.9 dB CNR = 7.0 dB ENL = 213.7 dB SSIM = 0.68
Zhou, 2023 [53]	OCT2017 [29] and OCTID [54], 5620 B-scans (5000/ 600/20)	Pre-processing: crop	Transformer-based NLM	N2N, DRGAN, Den-mimic-net, Contourlet, BM3D, INLSM, NLM, OBNLM, PNLM	MSE + gradient	CNR = 15.7 dB SNR = 51.1 dB ENL = 23,787 dB
Kande, 2020 [55]	Duke [12], 28 B-Scans (10/18)	NA	SiameseGAN	MSBTD, MIFCN, Shared Encoder, WGAN U-Net, WGAN ResNet	MS-SSIM + perceptual	PSNR = 28.3 dB SSIM = 0.83 MSR = 4.2 CNR = 2.6 dB TP = 0.68 EP = 0.66
Qiu, 2020 [56]	Duke [12], 52 groups of 50 B-scans each (37/15)	Pre-processing: crop	DBPN	BM3D, Bicubic, NWSR, U-Net	MSE	PSNR = 31.3 dB RMSE = 0.027 MS-SSIM = 0.92
Zhou, 2021 [57]	Topcon [13] and Cirrus [15], 521 B-scans (512/9)	Pre-processing: registering and averaging images Augmentation: flip, scaling, rotation, non-rigid transformation	GAN with HRNet	NLM, STROLLR, DnCNN, DPDNN, Edge-cGAN, mini-cGAN	L1 + MSE + Adversarial	SNR = 40.4 dB CNR = 11.2 dB SSI = 0.09 EPI = 0.96
Ahmed, 2022 [58]	Duke [12], 18 B-scans (10/8)	NA	DnCNN	BM3D, Weiner, NLM	CNR + pixel difference	PSNR = 29.6 dB CNR = 11.5 dB ENL = 1196.6 dB

Keywords: MSE—Mean squared error; MAE—Mean absolute error; SNR—Signal-to-noise ratio; PSNR—Peak signal-to-noise ratio; CNR—Contrast-to-noise ratio; SSIM—Structural similarity index measure;MS-SSIM—Multi-scale structural similarity index measure; ENL—Equivalent number of looks; EPI—Edge preservation index; RMA-GAN—Realistic mixed attention GAN; SRGAN—Super-resolution GAN; EGAN—Enhanced GAN; SRCNN—Super-resolution CNN; SSR—Self Super-resolution; SRResNet—Super-resolution ResNet; WGAN—Wasserstein GAN; DeGAN—Denoising GAN; BCE—Binary Cross Entropy; MGAN—Multi-task generative adversarial network; HWT—Hyperanalytic Wavelet Transform; AG—Attention Gate; MKSF—Multi-kernel speckle filtering block; N2N—Neighbor2Neighbor; DRGAN—Disentangled representation generative adversarial network; NLM—Nonlocal means.

## Supplementary Materials

The following supporting information can be downloaded at: https://www.mdpi.com/article/10.3390/jimaging10040086/s1, Table S1: List of excluded studies and the reasons; Table S2: Risk-of-bias assesment of papers reviewed.

## Abbreviations

MSE—Mean squared error; SNR—Signal-to-noise ratio; PSNR—Peak signal-to-noise ratio; CNR—Contrast-to-noise ratio; SSIM—Structural similarity index measure; ENL—Equivalent number of looks; BCE—Binary Cross Entropy; MGAN—Multi-task generative adversarial network; HWT—Hyperanalytic Wavelet Transform; AG—Attention Gate; MKSF—Multi-kernel speckle filtering block; N2N—Neighbor2Neighbor; DRGAN—Disentangled representation generative adversarial network; NLM—Nonlocal means; EPI—Edge preservation index; RMA-GAN—Realistic mixed attention GAN; SRGAN—Super-resolution GAN; EGAN—Enhanced GAN; SRCNN—Super-resolution CNN; SSR—Self Super-resolution; SRResNet—Super-ResNet;WGAN—Wasserstein GAN; DeGAN—Denoising GAN; BCE—Binary Cross Entropy; MGAN—Multi-task generative adversarial network. MSUN—Multi-scale U-Net.
