# Supplementary material for: Denoising of Optical Coherence Tomography Images in Ophthalmology Using Deep Learning: A Systematic Review
Source: J Imaging. 2024 Apr 1;10(4):86. doi: 10.3390/jimaging10040086 (PMC11050869; doi:10.3390/jimaging10040086)
Supplement: Supplementary file 1 [file jimaging-10-00086-s001.zip › jimaging-2913920-supplementary.pdf]

Denoising of Optical Coherence Tomography Images in Ophthalmology using deep learning: A systematic review

H. Ahmed, Q. Zhang, R. Donnan and A. Alomainy

Table S1

TABLE S1: LIST OF EXCLUDED STUDIES AND THE REASONS

Author, Year	Title	Reason
Dong, 2020 [1]	Optical coherence tomography image denoising using a generative adversarial network with speckle modulation	The dataset and the splitting procedure were not explained
Liu, 2021 [2]	Axial and horizontal registration guided speckle suppression in single-line HD mode for retinal optical coherence tomography images	Did not use any deep learning approaches
Cheng, 2021 [3]	Robust three-dimensional registration on optical coherence tomography angiography for speckle reduction and visualization	
Cheong, 2020 [4]	DeshadowGAN: A Deep Learning Approach to Remove Shadows from Optical Coherence Tomography Images	The dataset and the splitting procedure were not explained
Liu, 2017 [5]	Patch-based denoising method using low-rank technique and targeted database for optical coherence tomography image	Did not use any deep learning approaches
Daneshmand, 2021 [6]	Reconstruction of Optical Coherence Tomography Images Using Mixed Low Rank Approximation and Second Order Tensor Based Total Variation Method	
Samieinasab, 2020 [7]	Multivariate Statistical Modeling of Retinal Optical Coherence Tomography	
Hu, 2020 [8]	Noise Redistribution and 3D Shearlet Filtering for Speckle Reduction in Optical Coherence Tomography	
Fang, 2012 [9]	Sparsity based denoising of spectral domain optical coherence tomography images	
Chen, 2021 [10]	Optical coherence tomographic image denoising based on Chi-square similarity and fuzzy logic	
Liu, 2020 [11]	Using a dual-tree complex wavelet transform for denoising an optical coherence tomography angiography blood vessel image	
Gomez-Valverde, 2021 [12]	Adaptive compounding speckle-noise-reduction filter for optical coherence tomography images	
Esmaeili, 2020 [13]	Three-dimensional curvelet-based dictionary learning for speckle noise removal of optical coherence tomography	
Yu, 2022 [14]	A noise statistical distribution analysis-based two-step filtering mechanism for optical coherence tomography image despeckling	
Smitha, 2021 [15]	A nonlocal deep image prior model to restore optical coherence tomographic images from gamma distributed speckle noise	The dataset and the splitting procedure were not explained
Chen, 2021 [16]	Fusion denoising algorithm of optical coherence tomography image based on point-estimated and block-estimated	Did not use any deep learning approaches

Fan, 2020 [17]	OCT Image Restoration Using Non-Local Deep Image Prior	The outcome of the model was not adequately reported
Jorjandi, 2021 [18]	Statistical modeling of retinal optical coherence tomography using the Weibull mixture model	Did not use any deep learning approaches
Paul, 2019 [19]	Speckle Removal Using Diffusion Potential for Optical Coherence Tomography Images	
Wong, 2010 [20]	General Bayesian estimation for speckle noise reduction in optical coherence tomography retinal imagery	
Cheng, 2016 [21]	Speckle Reduction in 3D Optical Coherence Tomography of Retina by A-Scan Reconstruction	
Thapa, 2015 [22]	Reduction of speckle noise from optical coherence tomography images using multi-frame weighted nuclear norm minimization method	
Bian, 2015 [23]	Multiframe denoising of high-speed optical coherence tomography data using interframe and intraframe priors	
Cheng, 2015 [24]	Speckle reduction in optical coherence tomography by matrix completion using bilateral random projection	
Karri, 2016 [25]	Simultaneous reconstruction and restoration of sparsely sampled optical coherence tomography image through learning separable filters for deep architectures	The outcome of the model was not adequately reported
Lv, 2018 [26]	Speckle noise reduction of multi-frame optical coherence tomography data using multi-linear principal component analysis	Did not use any deep learning approaches
Cheng, 2019 [27]	Pixel Reconstruction For Speckle Reduction In 3D Optical Coherence Tomography Of Retina	
Yoo, 2019 [28]	Adaptive Weighted Nuclear Norm Minimization for Removing Speckle Noise from Optical Coherence Tomography Images	
Husvogt, 2021 [29]	Maximum a posteriori signal recovery for optical coherence tomography angiography image generation and denoising	
Chitchian, 2012 [30]	Retinal optical coherence tomography image enhancement via shrinkage denoising using double-density dual-tree complex wavelet transform	
Kopriva, 2016 [31]	Enhanced low-rank + sparsity decomposition for speckle reduction in optical coherence tomography	
Hu, 2019 [32]	Selective retinex enhancement based on the clustering algorithm and block-matching 3D for optical coherence tomography images	
Huang, 2021 [33]	Image Noise Recognition Algorithm Based on Improved DenseNet	The outcome of the model was not adequately reported
Hu, 2022 [34]	Unsupervised denoising of retinal OCT with diffusion probabilistic model	Did not use any deep learning approaches
Yan, 2020 [35]	Speckle reduction of OCT via super resolution reconstruction and its application on retinal layer segmentation	
Gupta, 2022 [36]	A De-Speckling Framework for Optical Coherence Tomography Images	
Huang, 2020 [37]	Both speckle reduction and contrast enhancement for optical coherence tomography via sequential optimization in the logarithmic domain based on a refined Retinex model	
Hu, 2021 [38]	Noise reduction by adaptive-SIN filtering for retinal OCT images	
Li, 2021 [39]	Speckle noise removal based on structural convolutional neural networks with feature fusion for medical image	The dataset and the splitting procedure were not explained

P.S, 2022 [40]	Despeckling of OCT images using DT-CWT based fusion technique	Did not use any deep learning approaches
Amini, 2019 [41]	Speckle Noise Reduction and Enhancement for OCT Images	
Yu, 2021 [42]	A two-step filtering mechanism for speckle noise reduction in OCT images	
Guo, 2020 [43]	Deep OCT image compression with convolutional neural networks	The dataset and the splitting procedure were not explained and the outcome of the model was not adequately reported
Wu, 2020 [44]	Cooperative Low-Rank Models for Removing Stripe Noise From OCTA Images	Did not use any deep learning approaches
Das, 2021 [45]	A diagnostic information based framework for super-resolution and quality assessment of retinal OCT images	
Schirrmacher, 2017 [46]	QuaSI: Quantile Sparse Image Prior for Spatio-Temporal Denoising of Retinal OCT Data	
Huang, 2021 [47]	Cluster-based filtering framework for removing speckles with structural protection in OCT images	
Yoo, 2020 [48]	CycleGAN-based deep learning technique for artifact reduction in fundus photography	The dataset and the splitting procedure were not explained and the outcome of the model was not adequately reported
Dong, 2020 [49]	Optical coherence tomography image de-noising using a generative adversarial network with speckle modulation	The dataset and the splitting procedure were not explained
Xu, 2021 [50]	Enhanced Visualization of Retinal Microvasculature via Deep Learning on OCTA Image Quality	The dataset and the splitting procedure were not explained and the outcome of the model was not adequately reported
Menon, 2019 [51]	A Novel Deep Learning Approach for the Removal of Speckle Noise from Optical Coherence Tomography Images Using Gated Convolution–Deconvolution Structure	The dataset and the splitting procedure were not explained
Gisbert, 2020 [52]	Self-supervised Denoising via Diffeomorphic Template Estimation: Application to Optical Coherence Tomography	The dataset and the splitting procedure were not explained and the outcome of the model was not adequately reported

Table S2

TABLE S2: RISK-OF-BIAS ASSESSMENT OF PAPERS REVIEWED

Author, Year	Reference	D1	D2	D3	D4
Devalla, 2019	53				

Cheong, 2021	54				
Tian, 2020	55				
Hu, 2020	56				
Akter, 2020	57				
Halupka, 2018	58				
Wei, 2018	59				
Chen, 2020	60				
Gour, 2020	61				
Hassan, 2021	62				
Ma, 2018	63				
Guo, 2020	64				
Qiu, 2020	65				
Huang, 2021	66				
Halupka, 2018	67				
Qiu, 2021	68				
Abassi, 2019	69				
Kande, 2020	70				
Qiu, 2020	71				
Shi, 2019	72				
Huang, 2020	73				
Yu, 2018	74				
Tajmirriahi, 2021	75				
Sengupta, 2021	76				
Mehdizadeh, 2021	77				
Cai, 2018	78				
Zhou, 2022	79				
Anoop, 2021	80				
Fu, 2021	81				

Wang, 2021	82				
Zhou, 2022	83				
Zhou, 2021	84				
Wu, 2021	85				
Das, 2020	86				
Huang, 2019	87				
Ge, 2022	88				
Ma, 2022	89				
Xie, 2022	90				
Xie, 2023	91				
Ahmed, 2022	92				
Ahmed, 2022	93				
Ahmed, 2022	94				
Zhou, 2023	95				

References

[1] Z. Dong, G. Liu, G. Ni, J. Jerwick, L. Duan, C. Zhou. Optical coherence tomography image denoising using a generative adversarial network with speckle modulation. *Journal of Biophotonics.* 2020:13; e201960135.

[2] M. Liu, X. Chen, B. Wang. Axial and horizontal registration guided speckle suppression in single-line HD mode for retinal optical coherence tomography images. Optics Communications. 2021:487;126807.

[3] Y. Cheng, Z. Chu, R. K. Wang. Robust three-dimensional registration on optical coherence tomography angiography for speckle reduction and visualization. *Quant Imaging Med Surg.* 2021:11;879–894.

[4] H. Cheong, S. K. Devalla, T. H. Pham, L. Zhang, M. J. A. Girard. DeshadowGAN: A Deep Learning Approach to Remove Shadows from Optical Coherence Tomography Images. *Translational Vision Science & Technology.* 2020:9;23-.

[5] X. Liu, Z. Yang, J. Wang, J. Liu, K. Zhang, W. Hu. Patch-based denoising method using low-rank technique and targeted database for optical coherence tomography image. *Journal of Medical Imaging*. 2017:4;014002.

[6] P. G. Daneshmand, A. Mehridehnavi, H. Rabbani. Reconstruction of Optical Coherence Tomography Images Using Mixed Low Rank Approximation and Second Order Tensor Based Total Variation Method. *IEEE Transactions on Medical Imaging.* 2021:40;865-878.

[7] M. Samieinasab, Z. Amini, H. Rabbani. Multivariate Statistical Modeling of Retinal Optical Coherence Tomography. *IEEE Transactions on Medical Imaging.* 2020:39;3475-3487.

[8] Y. Hu, J. Yang, J. Cheng, J. Liu. Noise Redistribution and 3D Shearlet Filtering for Speckle Reduction in Optical Coherence Tomography. *IEEE 17th International Symposium on Biomedical Imaging (ISBI).* 2020;1565-1569.

[9] L. Fang, S. Li, Q. Nie, J. A. Izatt, C. A. Toth and S. Farsiu, Sparsity based denoising of spectral domain optical coherence tomography images. *Biomedical Optics Express*. 2012:3;927-942.

[10] H. Chen, S. Fu, H. Wang. Optical coherence tomographic image denoising based on Chi-square similarity and fuzzy logic. *Optics & Laser Technology.* 2021:143;107298.

[11] H. Liu, S. Lin, C. Ye, D. Yu, J. Qin, L. An. Using a dual-tree complex wavelet transform for denoising an optical coherence tomography angiography blood vessel image. *OSA Continuum.* 2020:3;2630-2645.

[12] J. J. Gomez-Valverde, C> Sinz, E. A. Rank, Z. Chen, A. Santos, W. Drexler, M. J. Ledesma-Carbayo. Adaptive compounding speckle-noise-reduction filter for optical coherence tomography images. *Journal of Biomedical Optics.* 2021:26;065001.
[13] M. Esmaeili, A. M. Dehnavi, F. Hajizadeh, H. Rabbani. Three-dimensional curvelet-based dictionary learning for speckle noise removal of optical coherence tomography. *Biomedical Optics Express.* 2020:11;586-608.
[14] X. Yu, C. Ge, M. Li, J. Chen, P. P. Shum. A noise statistical distribution analysis-based two-step filtering mechanism for optical coherence tomography image despeckling. *Laser Physics Letters.* 2022:19;075601.
[15] A. Simtha and P. Jidesh. A nonlocal deep image prior model to restore optical coherence tomographic images from gamma distributed speckle noise. *Journal of Modern Optics.* 2021:68;1002-1017.
[16] H. Chen. Fusion denoising algorithm of optical coherence tomography image based on point-estimated and block-estimated. *Optik.* 2021:225;165864.
[17] W. Fan, H. Yu, T. Chen, S. Ji. OCT Image Restoration Using Non-Local Deep Image Prior. *Electronics.* 2020:9;784.
[18] S. Jorjandi, Z. Amini, G. Plonka, H. Rabbani. Statistical modeling of retinal optical coherence tomography using the Weibull mixture model. *Biomedical Optics Express.* 2021:12;5470-5488.
[19] A. Paul, D. P. Mukherjee, S. T. Acton. Speckle Removal Using Diffusion Potential for Optical Coherence Tomography Images. *IEEE Journal of Biomedical and Health Informatics.* 2019:23;264-272.
[20] A. Wong, A. Mishra, K. Bizheva, D. A. Clausi. General Bayesian estimation for speckle noise reduction in optical coherence tomography retinal imagery. *Optics Express.*2010:18;8338-8352.
[21] J. Cheng, D. Tao, Y. Quan, D. W. K. Wong, G. C. M. Cheung, M. Akiba, J. Liu. Speckle Reduction in 3D Optical Coherence Tomography of Retina by A-Scan Reconstruction. *IEEE Transactions on Medical Imaging.* 2016:35;2270-2279.
[22] D. Thapa, K. Raahemifar, V. Lakshminarayanan. Reduction of speckle noise from optical coherence tomography images using multi-frame weighted nuclear norm minimization method. *Journal of Modern Optics.* 2015:62;1856-18864.
[23] L. Bian, J. Suo, F. Chen, Q. Dai. Multiframe denoising of high-speed optical coherence tomography data using interframe and intraframe priors. *Journal of Biomedical Optics.* 2015:20;036006.
[24] J. Cheng, L. Duan, D. W. K. Wong, M. Akiba, J. Liu. Speckle reduction in optical coherence tomography by matrix completion using bilateral random projection. *36th Annual International Conference of the IEEE Engineering in Medicine and Biology Society.* 2014;186-189.
[25] S. P. K. Karri, N. Garai, D. Nawn, S. Ghosh, D. Chakraborty, J. Chatterjee. Simultaneous reconstruction and restoration of sparsely sampled optical coherence tomography image through learning separable filters for deep architectures. *IEEE Students' Technology Symposium (TechSym).* 2016;52-55.
[26] H. Lv, S. Fu, C. Zhang, L. Zhai. Speckle noise reduction of multi-frame optical coherence tomography data using multi-linear principal component analysis. *Optics Express.* 2018:26;11804-11818.
[27] J. Cheng, Y. Zhao, Y. Hu, J. Liu. Pixel Reconstruction For Speckle Reduction In 3D Optical Coherence Tomography Of Retina. *IEEE 16th International Symposium on Biomedical Imaging (ISBI).* 2019;1424-1427.
[28] S.Y. Yoo, Z. Wang, J. M. Seo. Adaptive Weighted Nuclear Norm Minimization for Removing Speckle Noise from Optical Coherence Tomography Images. *41st Annual International Conference of the IEEE Engineering in Medicine and Biology Society (EMBC).* 2019; 2687-2690.
[29] L. Husvogt, S. B. Ploner, S. Chen, D. Stromer, J. Schottenhamml, A. Y. Alibhai, E. Moult, N. K. Waheed, J. G. Fujimoto, A. Maier. Maximum a posteriori signal recovery for optical coherence tomography angiography image generation and denoising. *Biomedical Optics Express.* 2021:12;55-68.
[30] S. Chitchian, M. A. Mayer, A. Boretsky, F. J. Kuijk, M. Motamedi. Retinal optical coherence tomography image enhancement via shrinkage denoising using double-density dual-tree complex wavelet transform. *Journal of Biomedical Optics.* 2012:17; 116009.
[31] I. Kopriva, F. Shi, X. Chen. Enhanced low rank + sparsity decomposition for speckle reduction in optical coherence tomography. *Journal of Biomedical Optics.* 2016:21;076008.
[32] Y. Hu, C. Tang, M. Xu, Z. Lei. Selective retinex enhancement based on the clustering algorithm and block-matching 3D for optical coherence tomography images. Applied Optics. 2019:58;9861-9869.
[33] M. Huang, L. Zeng, Y. Zhang, Y. Li, Z. Ni, D. Wu, S. Feng. Image Noise Recognition Algorithm Based on Improved DenseNet. *Web Information Systems and Applications.* 2021;455-467.
[34] D. Hu, Y. K. Tao, I. Oguz. Unsupervised denoising of retinal OCT with diffusion probabilistic model. *Medical Imaging 2022: Image Processing*. 2022:12032;1203206.
[35] Q. Yan, B. Chen, Y. Hu, J. Cheng, Y. Gong, J. Yang, J. Liu, Y. Zhao. Speckle reduction of OCT via super resolution reconstruction and its application on retinal layer segmentation. *Artificial Intelligence in Medicine.* 2020:106;101871.
[36] P. K. Gupta and F. Husain. A De-Speckling Framework for Optical Coherence Tomography Images. *Proceedings of Trends in Electronics and Health Informatics*. 2022;207-215.

[37] Z. Huang, C. Tang, M. Xu, Y. Shen, Z. Lei. Both speckle reduction and contrast enhancement for optical coherence tomography via sequential optimization in the logarithmic domain based on a refined Retinex model. *Applied Optics*. 2020:59;11087-11907.

[38] Y. Hu, J. Ren, J. Yang, R. Bai, J. Liu. Noise reduction by adaptive-SIN filtering for retinal OCT images. *Scientific Reports.* 2021:11;19498.

[39] D. Li, W. Yu, K. Wang, D. Jiang, Q. Jin. Speckle noise removal based on structural convolutional neural networks with feature fusion for medical image. *Signal Processing: Image Communication.* 2021:99;116500.

[40] A. P. S., V. P. Gopi, P. Palanisamy. Despeckling of OCT images using DT-CWT based fusion technique. *Optik.* 2022:263;169332.

[41] Z. Amini, R. Kafieh, H. Rabbani. Speckle Noise Reduction and Enhancement for OCT Images. *Retinal Optical Coherence Tomography Image Analysis.* 2019;39-72.

[42] X. Yu, C. Ge, Z. Fu, M. Z. Aziz, L. Liu. A two-step filtering mechanism for speckle noise reduction in OCT images. *IEEE 9th International Conference on Information, Communication and Networks (ICICN).* 2021;501-505.

[43] P. Guo, D. Li, X. Li. Deep OCT image compression with convolutional neural networks. *Biomedical Optics Express.* 2020:11;3543-3554.

[44] X. Wu, D. Gao, D. Borroni, S. Madhusudhan, Z. Jin, Y. Zheng. Cooperative Low-Rank Models for Removing Stripe Noise From OCTA Images. *IEEE Journal of Biomedical and Health Informatics.* 2020:24;3480-3490.

[45] V. Das, S. Dandapat, P. K. Bora. A diagnostic information-based framework for super-resolution and quality assessment of retinal OCT images. *Computerized Medical Imaging and Graphics*. 2021:94;101997.

[46] F. Schirrmacher, T. Köhler, L. Husvogt, J. G. Fujimoto, J. Hornegger, A. K. Maier. QuaSI: Quantile Sparse Image Prior for Spatio-Temporal Denoising of Retinal OCT Data. *Medical Image Computing and Computer-Assisted Intervention – MICCAI.* 2017;93-91.

[47] S Huang, C. Tang, M. Xu, Z. Lei. Cluster-based filtering framework for removing speckles with structural protection in OCT images. *Applied Physics B.* 2021:127;149.

[48] T. K. Yoo, J. Y. Choi, H. K. Kim. CycleGAN-based deep learning technique for artifact reduction in fundus photography. *Graefe's Archive for Clinical and Experimental Ophthalmology.* 2020:258;1631-1637.

[49] Z. Dong, G. Liu, G. Ni, J. Jerwick, L. Duan, C. Zhou. Optical coherence tomography image de-noising using a generative adversarial network with speckle modulation. *Biophotonics.* 2020:13;e201960135.

[50] Y. Xu, Y. Su, D. Hua, P. Heiduschka, W. Zhang, T. Cao, J. Liu, Z. Ji, N. Eter. Enhanced Visualization of Retinal Microvasculature via Deep Learning on OCTA Image Quality. *Dis Markers.* 2021;1373362.

[51] S. N. Menon, V. B. V. Reddy, A. Yeshwanth, B. N. Anoop, J. Rajan. A Novel Deep Learning Approach for the Removal of Speckle Noise from Optical Coherence Tomography Images Using Gated Convolution–Deconvolution Structure. *Proceedings of 3rd International Conference on Computer Vision and Image Processing.*2019;115-126.

[52] G. Gisbert, N. Dey, H. Ishikawa, J. Schuman, J. Fishbaugh, G. Gerig. Self-supervised Denoising via Diffeomorphic Template Estimation: Application to Optical Coherence Tomography. *Ophthalmic Medical Image Analysis (OMIA).* 2020;72-82.

[53] S.K. Devalla, G. Subramanian, T. H. Pham, X. Wang, S. Perera, T. A. Tun, T. Aung, L. Schmetterer, A. H. Thiery and M. J. A. Girard. A Deep Learning Approach to Denoise Optical Coherence Tomography Images of the Optic Nerve Head. *Scientific Reports.* 2019:9; 14454.

[54] H. Cheong, S. K. Devalla, T. Chuangsuwanich, T. A. Tun, X. Wang, T. Aung, L. Schmetterer, M. L. Buist, C. Boote, A. H. Thiery and M. A. Girard. OCT-GAN: single step shadow and noise removal from optical coherence tomography images of the human optic nerve head. *Biomedical optics express*. 2021:12;1482-1498.

[55] C. Tian, J. Yang, P. Li, S. Zhang and S. Mi. Retinal fundus image superresolution generated by optical coherence tomography based on a realistic mixed attention GAN. *Medical Physics.* 2022:49;3185-31998.

[56] D. Hu, J. D. Malone, Y. Atay, Y. K. Tao and I. Oguz. Retinal OCT Denoising with Pseudo-Multimodal Fusion Network. *OMIA 2020: Ophthalmic Medical Image Analysis*. 2020; 125-135.

[57] N. Akter, S. Perry, J. Fletcher, M. Simunovic, M. Roy. Automated Artifacts and Noise Removal from Optical Coherence Tomography Images Using Deep Learning Technique. *2020 IEEE Symposium Series on Computational Intelligence (SSCI).* 2020;2536-2542.

[58] K. Halupka, B. Antony. M. Lee, K. Lucy, R. Rai, H. Ishikawa, G. Wollstein, J. Schuman and R. Garnavi. Retinal optical coherence tomography image enhancement via deep learning. *Biomedical optics express*. 2018:9;6205-6221.

[59] X. Wei, X. Liu, A. Yu, T. Fu, D. Liu. Clustering-Oriented Multiple Convolutional Neural Networks for Optical Coherence Tomography Image Denoising. 11th International Congress on Image and Signal Processing, BioMedical Engineering and Informatics (CISP-BMEI). 2018:1-5.

[60] Z. Chen, Z. Zheng, H. Shen, Z. Zheng, P. Dai, P. Ouyang. DN-GAN: Denoising generative adversarial networks for speckle noise reduction in optical coherence tomography images. *Biomedical Signal Processing and Control.* 2020:55;101632.

[61] N. Gour and P. Khanna. Speckle denoising in optical coherence tomography images using residual deep convolutional neural network. *Multimedia Tools and applications.* 2020:79;15679-15695.

[62] M. J. Hasan, M. S. Alom, U. Fatema, M. F. Wahid. Deep Learning Based Retinal OCT Image Denoising using Generative Adversarial Network. *International Conference on Automation, Control and Mechatronics for Industry 4.0 (ACMI).* 2021;1-6.

[63] Y. Ma, X. Chen, W. Zhu, X. Cheng, D. Xiang and F. Shi. Speckle noise reduction in optical coherence tomography images based on edge-sensitive cGAN. *Biomedical Optics Express.* 2018:9;5129-5146.

[64] A. Guo, L. Fang, M. Qi, S. Li. Unsupervised Denoising of Optical Coherence Tomography Images With Nonlocal-Generative Adversarial Network. *IEEE Transactions on Instrumentation and Measurement.* 2020:70;1-12.

[65] B. Qiu, Z. Huang, X. Liu, X. Meng, Y. You, G. Liu, K. Yang, A. Maier, Q. Ren, Y. Lu. Noise reduction in optical coherence tomography images using a deep neural network with perceptually sensitive loss function. *Biomedical Optics Express.* 2020:11;817-830.

[66] Y. Huang, N. Zhang and Q. Hao. Real-time noise reduction based on ground truth free deep learning for optical coherence tomography. *Biomedical Optics Express.* 2021:12;2027-2040.

[67] K. J. Halupka, B. J. Antony, M. H. Lee, K. A. Lucy, R. S. Sai, H. Ishikawa. G. Wollstein, J. S. Schuman, R. Garnavi. Retinal optical coherence tomography image enhancement via deep learning. *Biomedical Optics Express.* 2018:9;6205-6221.

[68] B. Qiu, S. Zeng, X. Meng, Z. Jiang, Y. You, M. Geng, Z. Li, Y. Hu, Z. Huang, C. Zhou, Q. Ren, Y. Lu. Comparative study of deep neural networks with unsupervised Noise2Noise strategy for noise reduction of optical coherence tomography images. *Journal of Biophotonics.* 2021:14; e202100151.

[69] A. Abbasi, A. Monadjemi, L. Fang, H. Rabbanni, Y. Zhang. Three-dimensional optical coherence tomography image denoising through multi-input fully convolutional networks. *Computers in Biology and Medicine.* 2019:108;1-8.

[70] N. A. Kande, R. Dakhane, A. Dukkipati, P. K. Yalavarthy. SiameseGAN: A Generative Model for Denoising of Spectral Domain Optical Coherence Tomography Images. *IEEE Transactions on Medical Imaging.* 2021:40;180-192.

[71] B. Qiu, Y. You, Z. Huang, X. Meng, Z. Jiang, C. Zhou, G. Liu, K. Yang, Q. Ren, Y. Lu. N2NSR-OCT: Simultaneous denoising and super-resolution in optical coherence tomography images using semisupervised deep learning. *Journal of Biophotonics.* 2020:14; e202000282.

[72] F. Shi, N. Cai, Y. Gu, D. Hu, Y. Ma, Y. Chen and X. Chen. DeSpecNet: a CNN-based method for speckle reduction in retinal optical coherence tomography images. *Physics in Medicine & Biology.* 2019:64;175010.

[73] Y. Huang, W. Xia, Z. Lu, Y. Liu, H. Chen, J. Zhou, L. Fang, Y. Zhang. Noise-Powered Disentangled Representation for Unsupervised Speckle Reduction of Optical Coherence Tomography Images. *IEEE Transactions on Medical Imaging.* 2021:40;2600-2614.

[74] A. Yu, X. Liu, X. Wei, T. Fu, D. Liu. Generative Adversarial Networks with Dense Connection for Optical Coherence Tomography Images Denoising. *11th International Congress on Image and Signal Processing, BioMedical Engineering and Informatics (CISP-BMEI).* 2019;1-5.

[75] M. Tajmirriahi, R. Kafieh, Z. Amini, H. Rabbani. A Lightweight Mimic Convolutional Auto-Encoder for Denoising Retinal Optical Coherence Tomography Images. *IEEE Transactions on Instrumentation and Measurement.* 2021:70;1-8.

[76] S. Sengupta, A. Singh, V. Lakshminarayanan. EdgeWaveNet: edge aware residual wavelet GAN for OCT image denoising. *Medical Imaging 2021: Imaging Informatics for Healthcare, Research, and Applications.*2021:11601;110-115.

[77] M. Mehdizadeh, C. MacNish, D. Xiao, D. Alonso-Caneiro, J. Kugelman, M. Bennamoun. Deep feature loss to denoise OCT images using deep neural networks. *Biomedical Optics.* 2021:26;046003.

[78] N. Cai, F. Shi, D. Hu, Y. Chen. A ResNet-based universal method for speckle reduction in optical coherence tomography images. Proc. IEEE Int. Symp. Biomed. Imaging (ISBI).2018.

[79] Q. Zhou, M. Wen, M. Ding, and X. Zhang. Unsupervised despeckling of optical coherence tomography images by combining cross-scale CNN with an intra-patch and inter-patch-based transformer. Optics Express. 2022:30;18800-18820.

[80] B.N. Anoop, K. S. Kalmady, A. Udathu, V. Siddharth, G. N. Girish, A. R. Kothari, J. Rajan. A cascaded convolutional neural network architecture for despeckling OCT images. Biomedical Signal Processing and Control. 2021:66;102463.

[81] Z. Fu, X. Yu, C. Ge, M. Z. Aziz, L. Liu. ADGAN: An Asymmetric Despeckling Generative Adversarial Network for Unpaired OCT Image Speckle Noise Reduction. *IEEE 6th Optoelectronics Global Conference (OGC).*2021;212-216.

[82] M. Wang, W. Zhu, K. Yu, Z. Chen, F. Shi, X. Chen. Semi-Supervised Capsule cGAN for Speckle Noise Reduction in Retinal OCT Images. *IEEE Transactions on Medical Imaging.* 2021:40;1168-1183.

[83] Y. Zhou, K. Yu, M. Wang, Y. Ma, Y. Peng, Z. Chen, W. Zhu, F. Shi, X. Chen. Speckle Noise Reduction for OCT Images Based on Image Style Transfer and Conditional GAN. *IEEE Journal of Biomedical and Health Informatics.* 2022:26;139-150.

[84] Y. Zhou, J. Li, M. Wang, W. Zhu, Y. Peng, Z. Chen, L. Wang, T. Wang, C. Yao, T. Wang and X. Chen. High-Resolution Hierarchical Adversarial Learning for OCT Speckle Noise Reduction. *MICCAI 2021: Medical Image Computing and Computer Assisted Intervention.* 2021;372-381.

[85] M. Wu, W. Chen, Q. Chen, H. Park. Noise Reduction for SD-OCT Using a Structure-Preserving Domain Transfer Approach. *IEEE Journal of Biomedical and Health Informatics.* 2021:25;3460-3472.

[86] V. Das, S. Dandapat, P. K. Bora. Unsupervised Super-Resolution of OCT Images Using Generative Adversarial Network for Improved Age-Related Macular Degeneration Diagnosis. *IEEE Sensors Journal.* 2020:20;8746-8756.

[87] Y. Huang, Z. Lu, Z. Shao, M. Ran, J. Zhou, L. Fang, Y. Zhang. Simultaneous denoising and super-resolution of optical coherence tomography images based on generative adversarial network. *Optics Express.* 2019:27;12289-12307.

[88] C. Ge, X. Yu, M. Li, J. Mo. Self-Supervised Denoising of single OCT image with Self2Self-OCT Network. *IEEE 7th Optoelectronics Global Conference (OGC)*. 2022:200-204.

[89] Z. Ma, Q. Xie, F. Fan, J. Zhu. DSGAN: a generative model for speckle noise reduction in retinal optical coherence tomography images. *Proc. SPIE 12320, Optics in Health Care and Biomedical Optics XII*. 2022:123201H.

[90] K. Xie, M. Luo, H. Chen, M. Yang, Y. He, P. Liao and Y. Zhang. Speckle denoising of optical coherence tomography image using residual encoder–decoder CycleGAN. *Signal, Image and Video Processing*. 2022:1-13.

[91] Q. Xie, Z. Ma, L. Zhu, F. Fan, X. Meng, X. Gao and J. Zhu. Multi-task generative adversarial network for retinal optical coherence tomography image denoising. *Physics in Medicine & Biology*. 2022:68;045002.

[92] H. Ahmed, Q. Zhang, R. Donnan and A. Alomainy. Unsupervised Region-Based Denoising for Optical Coherence Tomography Framework. *International Conference on Computational Intelligence and Applications (ICCIA).* 2022:267-273.

[93] H. Ahmed, Q. Zhang, R. Donnan and A. Alomainy. Framework of Unsupervised based Denoising for Optical Coherence Tomography. *International Conference on Biomedical Signal and Image Processing (ICBIP*). 2022:19-24.

[94] H. Ahmed, Q. Zhang, R. Donnan and A. Alomainy. Attention Based Speckle Reduction for Optical Coherence Tomography in Ophthalmology and Dentistry. *International Congress on Image and Signal Processing, BioMedical Engineering and Informatics (CISP-BMEI).* 2022:1-6.

[95] Q. Zhou, M. Wen, B. Yu, C. Lou, M. Ding, X. Zhang. Self-supervised transformer based non-local means despeckling of optical coherence tomography images. *Biomedical Signal Processing and Control.* 2023:80;104348.
